# Emulsion Gel Stabilized by Young Bamboo Culm (*Phyllostachys edulis*) Fiber–Soy Protein Composite: Ball-Milling Effects on Stability and Lipid Digestion

**DOI:** 10.3390/foods15193460

**Published:** 2026-09-28

**Authors:** Zixuan Zhou, Chenlu Luo, Shuang Bi, Xian Cui, Xiaoxiao Song

**Affiliations:** 1 State Key Laboratory of Food Science and Resources, Engineering Research Center for Biomass Conversion, Ministry of Education, Nanchang University, Nanchang 330047, Chinacuixian@ncu.edu.cn (X.C.); 2 School of Food and Health, Beijing Technology & Business University, Beijing 100048, China; bishuang@btbu.edu.cn

**Keywords:** bamboo culm dietary fiber, ball milling, in vitro lipid digestion, interfacial stabilization

## Abstract

This study aimed to valorize discarded moso bamboo (*Phyllostachys edulis*) culm tips, a fiber-rich food processing by-product, into a clean-label structuring ingredient with controlled lipid digestibility. Bamboo culm tip insoluble dietary fiber (BIDF) was ball-milled for 0 to 12 h and combined with soy protein isolate (SPI) to stabilize camellia oil-in-water fiber-network-stabilized emulsions at an oil fraction of 0.7; lipolysis was evaluated under INFOGEST 2.0. Ball milling for 9 h reduced BIDF to sub-10 μm particles and raised its three-phase contact angle from approximately 98° to 105° while preserving the cellulose I crystal form, and this milled fiber (SM-BIDF) was selected for emulsification. At 0.1% (*w*/*v*) SM-BIDF, emulsions showed the smallest droplet size (8.68 μm, vs. 18.4 μm for SPI-only), the highest |ζ|-potential (53.23 mV), and a gel network that remained stable for 14 days at 4 °C; this formulation also released the lowest free fatty acids (24.76% at 120 min), substantially below SPI-only controls. A concentration-dependent three-stage mechanism of interfacial reinforcement, network optimization, and excessive weakening was proposed to explain these observations. These results demonstrate that ball-milled bamboo culm tip IDF combined with SPI provides a sustainable dual-functional emulsifier that simultaneously stabilizes emulsions and retards lipid digestion.

## 1. Introduction

Dietary fiber (DF) is defined as carbohydrate polymers that are not hydrolyzed by endogenous digestive enzymes in the human small intestine, encompassing cellulose, hemicellulose, pectin, lignin, and oligosaccharides [1,2]. Agricultural and food processing by-products represent abundant and underutilized sources of dietary fiber, and their valorization into functional ingredients aligns with circular bioeconomy principles [3,4]. Among these by-products, the young culms of moso bamboo (*Phyllostachys edulis*), commonly known as bamboo shoots, are particularly noteworthy: their tender culm tips, which account for approximately 40% of the harvested weight, are typically discarded during industrial processing due to high lignification and coarse texture [5]. These culm tips are rich in dietary fiber (60–70% on a dry-weight basis), with IDF comprising 70–80% of the total fiber [6,7]. Adequate DF intake confers multiple health benefits, including regulation of gastrointestinal function, modulation of gut microbiota composition, enhancement of short-chain fatty acid production, and reduction in the risk of cardiovascular disease, type 2 diabetes, and colorectal cancer [1,8,9,10]. These health benefits originate from the functional groups of the fiber constituents: the abundant hydroxyl (–OH) groups of cellulose and hemicellulose underlie water-holding and glucose entrapment, the carboxyl (–COOH) groups of pectin confer cation-exchange and bile-acid binding capacity, and the phenolic hydroxyl and aromatic moieties of lignin provide hydrophobicity and lipid/cholesterol adsorption [11,12]. Bamboo culm DF, in particular, has been shown to exhibit hypolipidemic effects in hyperlipidemic rats, promote beneficial shifts in the gut microbiota, and possess favorable hydration and binding properties [13,14,15].

In the food industry, emulsifiers play an essential role in formulating stable oil-in-water systems. Compared with synthetic emulsifiers, natural food-grade emulsifiers are increasingly preferred owing to consumer demand for clean-label products and concerns regarding the potential health risks of chemical additives [16,17]. Insoluble dietary fiber (IDF) is particularly attractive in this context: being naturally derived and biodegradable, it can act as a clean-label thickener or stabilizer in food emulsions, potentially replacing synthetic additives such as polysorbates (E432–E436) while maintaining adequate emulsion stability [18,19,20]. Emulsions are commonly produced by high-energy methods such as high-shear homogenization, colloid milling, and high-pressure homogenization. Non-conventional routes, including ultrasonication, microfluidization, membrane emulsification, and low-energy phase inversion, allow finer control over droplet size, though often with lower throughput or greater operational complexity [21,22]. For emulsions containing insoluble dietary fiber, high-pressure homogenization, high-shear homogenization, and microfluidization are currently the most widely used methods [23]. Among emulsions, particle-stabilized systems—either by irreversible adsorption at the oil–water interface or by formation of a continuous-phase particle network—offer superior coalescence resistance, long-term stability, and the ability to modulate lipid digestion kinetics compared with conventional surfactant-stabilized emulsions [24,25]. Dietary fiber and cellulose-based particles have attracted substantial attention as food-grade particulate stabilizers owing to their natural abundance, biocompatibility, and biodegradability [26,27]. Because interfacial adsorption requires the particles to be far smaller than the droplets, realizing this stabilizing role depends on reducing the fiber down to the nanoscale. Accordingly, He et al. [28] converted bamboo culm water-insoluble DF into nanoscale filaments by high-pressure homogenization, which allowed these particles to adsorb at the oil–water interface and stabilize O/W emulsions for over one month. Lei et al. [29] likewise exploited the nanoscale dimensions of bamboo culm nanocellulose to obtain an efficient particulate stabilizer with outstanding coalescence resistance. These findings indicate that reducing bamboo culm-derived DF to the nanoscale enables interfacial particle stabilization; however, native IDF possesses a dense and compact structure, poor hydrophilicity, and limited interfacial activity, which severely restricts its direct application as an emulsifier [30,31].

To overcome these limitations, dietary fiber is commonly modified before use. The modification technologies reported to date fall into three categories: chemical methods (acid or alkaline hydrolysis, carboxymethylation, cross-linking), which effectively restructure the fiber but may leave reagent residues and conflict with the formulation of clean-label food products; enzymatic methods (cellulase, xylanase), which are selective but costly and difficult to control at industrial scale; and physical methods (ball milling, micronization, microfluidization, high-pressure homogenization), which modify the fiber without altering its native chemical structure and are therefore regarded as the most suitable routes for clean-label food applications [32,33]. Among the physical routes, ball milling is solvent-free, easy to operate, and its time-dependent action allows the degree of modification to be tuned [34]. Accordingly, two principal strategies were explored in this study, the first being mechanical modification by ball milling, which reduces the particle size, increases the specific surface area, disrupts crystalline regions, and exposes previously buried functional groups [35,36]. Studies on wheat bran, pomelo peel, bamboo culm shell, and coffee husk IDF have demonstrated that ball milling alters morphology, crystallinity, hydration properties, and surface wettability in a time-dependent manner [37,38]. Niu et al. [39] reported that ball milling significantly reduced the particle size of IDF from grain brans and altered their crystallinity and hydration properties. Ke et al. [34] demonstrated that ball milling improved the interfacial properties of Polygonatum sibiricum IDF, with the contact angle reaching approximately 88.7°, suitable for Pickering stabilization. The second strategy is the construction of protein–polysaccharide composite systems, where proteins provide interfacial adsorption capacity through their amphiphilic nature while polysaccharides reinforce the continuous-phase network [40,41]. Soy protein isolate (SPI), a widely available plant protein with excellent emulsifying properties, has been extensively used in such composites [42]. Li et al. [43] showed that cellulose nanofiber–SPI composites synergistically enhanced the stability of Pickering emulsions. Zhang et al. [44] reported that bacterial cellulose–SPI interactions at the oil–water interface significantly influenced both emulsion stability and in vitro digestibility. Ashaolu and Zhao [45] fabricated okara IDF–SPI conjugates as an effective composite structuring agent. Although these strategies have shown promise, systematic research on ball-milled bamboo culm tip IDF and its composite system with SPI for a composite structuring agent remains limited.

Therefore, the present study aimed to (1) investigate the effects of ball-milling time (0, 3, 6, 9, and 12 h) on the structural characteristics, physicochemical properties, and wettability of BIDF; (2) evaluate the feasibility of using SM-BIDF (9 h ball-milled) and SPI as a composite Pickering stabilizer for camellia oil; and (3) elucidate the concentration-dependent relationships among SM-BIDF level, emulsion stability, and in vitro lipid digestion kinetics. The resulting formulation is intended as a clean-label structuring ingredient for oil-in-water emulsions and as a delivery system for lipophilic nutrients with a moderated rate of lipid digestion.

## 2. Materials and Methods

### 2.1. Materials

Fresh moso bamboo (*Phyllostachys edulis*) culm tips were obtained from a local bamboo culm processing facility in Jiangxi Province, China. The culm tips were washed, sliced, dried at 50 °C in a thermostatic forced-air drying oven (ZXRD-B5210, Shanghai Zhicheng, Shanghai, China), and ground to pass a 60-mesh sieve. Soy protein isolate (SPI, protein content ≥ 90%), bile salts (CAS 8008-63-7), hydrochloric acid (CAS 7647-01-0), and simulated gastric fluid (with enzymes) were purchased from Shanghai Yuanye Bio-Technology Co., Ltd. (Shanghai, China). Camellia oil (Camellia oleifera Abel.) was purchased from a local camellia oil factory in Ji’an, Jiangxi, China; the oil contained 99.8 g fat per 100 g, with approximately 78.5% monounsaturated, 10.4% polyunsaturated, and 10.9% saturated fatty acids, and met the requirements of Grade 1 press-extracted camellia oil of GB/T 11765 [46] (acid value ≤ 1.0 mg KOH/g; moisture and volatile matter ≤ 0.10%). Its refractive index and relative density were approximately 1.46 (25 °C) and 0.91 g/cm^3^. The total dietary fiber assay kit (K-TDFR-200A) was obtained from Megazyme (Bray, Ireland) and used for the determination of insoluble dietary fiber (IDF) and soluble dietary fiber (SDF).Pancreatic lipase (P7000, ≥250 U/mg, CAS 9001-62-1) was purchased from Sigma-Aldrich (St. Louis, MO, USA). Pancreatin (8x USP, CAS 8049-47-6) and boric acid (CAS 10043-35-3) were purchased from Shanghai Macklin Biochemical Co., Ltd. (Shanghai, China). Acetone (CAS 67-64-1), ethanol (CAS 64-17-5), concentrated sulfuric acid (CAS 7664-93-9), and petroleum ether (CAS 8032-32-4) were purchased from Xilong Scientific Co., Ltd. (Guangzhou, China). Potassium bromide (CAS 7758-02-3) was purchased from Shanghai Aladdin Biochemical Technology Co., Ltd. (Shanghai, China). All other chemicals were of analytical grade.

### 2.2. Extraction and Ball-Milling Modification of BIDF

IDF was extracted from bamboo culm tip powder using an enzymatic–gravimetric method following the AOAC 991.43 protocol [47] with slight modifications. Briefly, the defatted powder was sequentially treated with heat-stable alpha-amylase, protease, and amyloglucosidase. The insoluble residue was washed with 95% ethanol and deionized water, dried, and sieved (60 mesh) to obtain native BIDF (N-BIDF).

Ball-milling modification followed the method described by Gao et al. [48] with modifications. N-BIDF powder (5 g) was milled in a planetary ball mill (MQ-3SP04, Nanjing Nanda Instrument Co., Ltd., Nanjing, China) with a 250 mL agate jar containing agate balls (5 mm, 40 balls; 10 mm, 16 balls) at a ball-to-powder mass ratio of approximately 8:1. Milling was conducted at 25 Hz in forward rotation mode, alternating 30 min of grinding with 0.02 min of rest; no active temperature control was applied, and the jar remained near ambient temperature throughout milling. Samples were collected after 3, 6, 9, and 12 h of cumulative milling and designated as BIDF (3), BIDF (6), BIDF (9), and BIDF (12), respectively [25]. BIDF (9) was selected for subsequent emulsion studies and is referred to as SM-BIDF.

### 2.3. Characterization of Ball-Milled BIDF

#### 2.3.1. Scanning Electron Microscopy (SEM)

Morphological characteristics were observed using a cold field-emission scanning electron microscope (SU8100, Hitachi High-Technologies Corporation, Tokyo, Japan) [39]. Samples were mounted on aluminum stubs, sputter-coated with gold (approximately 10 nm), and imaged at an accelerating voltage of 3 kV. Representative micrographs were acquired at magnifications of 500×, 2000×, and 5000×.

#### 2.3.2. X-Ray Diffraction (XRD)

XRD patterns were recorded using an X-ray diffractometer (D8 Advance, Bruker, Karlsruhe, Germany) with Cu Kalpha radiation (lambda = 0.15406 nm) at 40 kV and 30 mA [49]. Samples were scanned over a 2-theta range of 5–50 degrees at 2 degrees/min. The relative crystallinity index (CI) was calculated using MDI Jade 9.0 software [38].

#### 2.3.3. Fourier Transform Infrared Spectroscopy (FTIR)

FTIR spectra were recorded on a Nicolet 5700 spectrometer (Thermo Fisher Scientific, Waltham, MA, USA) using the KBr pellet method [39]. Samples were mixed with spectroscopic-grade KBr at approximately 1:100, ground in an agate mortar, and pressed into transparent pellets. Spectra were acquired in the range of 400–4000 cm^−1^ with a resolution of 4 cm^−1^ and 64 scans per sample.

#### 2.3.4. Three-Phase Contact Angle

BIDF wettability was evaluated by measuring the three-phase contact angle (theta) using a drop shape analyzer (OCA-50, Dataphysics, Filderstadt, Germany) [48]. BIDF powder was compressed into a flat tablet (approximately 13 mm diameter, 1 mm thickness) under 30 MPa for 2 min. The tablet was immersed in camellia oil, and a 3 uL deionized water droplet was deposited onto the tablet surface. Images were captured after 2 min of equilibration, and theta was determined by Young–Laplace fitting.

#### 2.3.5. Water-Holding and Oil-Holding Capacity

WHC was determined by dispersing 0.2 g of BIDF in 5 mL of deionized water, shaking at 25 ± 2 °C for 1 h, centrifuging at 4500 rpm for 15 min in a centrifuge (Primo R, Thermo Fisher Scientific, USA), and weighing the wet residue [39]. OHC was measured using the same procedure with camellia oil, using 0.1 g of each sample and centrifugation at 4000 rpm for 15 min [48].

### 2.4. Preparation of SPI-BIDF Composite Emulsions

SPI stock solution (4% *w*/*v*) was prepared by dispersing SPI powder in deionized water, adjusting the pH to 7.0 ± 0.05 using a pH meter (PHS-3G, INESA Scientific Instrument Co., Ltd., Shanghai, China), and hydrating overnight at 4 °C [43]. SM-BIDF suspensions (0, 0.1%, 0.2%, 0.4%, 0.6%, and 0.8% *w*/*v*) were prepared by dispersing SM-BIDF powder in deionized water, stirring magnetically for at least 4 h, and sonicating for 20 min using an ultrasonic cleaner (SK7200H, Shanghai Codyson Ultrasonic Instrument Co., Ltd., Shanghai, China).

The SPI-BIDF composite emulsifier was prepared by mixing the 4% SPI stock with SM-BIDF suspensions at a 1:1 (*v*/*v*) ratio (the final SPI, SM-IBDF and oil volume fraction concentrations are exhibited in Table 1). The mixture was stirred at 500 rpm for 30 min at 25 ± 2 °C using a magnetic stirrer (MS-H280-Pro, DRAGONLAB, Beijing, China) [44,45].

Emulsions were prepared by adding camellia oil at an oil phase volume fraction (φ, defined as the volume of camellia oil divided by the total emulsion volume) of 0.7, followed by high-speed shearing at 15,000 rpm for 2 min using a high-shear homogenizer (XHF-DY, Ningbo Xinzhi Biotechnology, Ningbo, China) [28,50]. Potassium sorbate (0.02% *w*/*v*) was added as an antimicrobial agent. All emulsions were prepared in triplicate.

### 2.5. Characterization of Emulsions

#### 2.5.1. Emulsifying Activity Index (EAI)

The emulsifying activity index (EAI) reflects the interfacial area stabilized per unit mass of protein, i.e., the ability of the emulsifier to adsorb at the oil–water interface and form an interfacial film. EAI was determined following the method of Li et al. [43] with modifications. Immediately after emulsion preparation, 50 uL fresh emulsion was diluted with 5 mL 0.1% (*w*/*v*) SDS solution. Absorbance at 500 nm was measured using a UV-Vis spectrophotometer (UV-9000, Shanghai Metash Instruments Co., Ltd., Shanghai, China) with 0.1% SDS as the blank. EAI was calculated using Equation (1):

(1) EAI = (2 × 2.303 × A0 × N)/(C × phi × 10,000)

where A0 is the absorbance, N is the dilution factor (100), C is the protein concentration (g/mL), and phi is the oil volume fraction (0.5) [49]. Measurements were performed in triplicate.

#### 2.5.2. Droplet Size and ζ-Potential Measurements

Droplet-size distribution was measured using a Mastersizer 3000 laser diffraction particle size analyzer (Malvern Panalytical, Worcestershire, UK) with refractive indices of 1.45 (droplet) and 1.33 (continuous phase) [50]. The surface-weighted mean diameter (D_3,2_) was used to characterize droplet size. ζ-potential was determined using a Zetasizer Pro (Malvern Panalytical, Worcestershire, UK) after 50-fold dilution with ultrapure water at 25 °C [49].

The Span index was calculated using Equation (2):

(2) Span index = (D_90_ − D_10_)/D_50_

where D_10_, D_50_ and D_90_ are the droplet diameters below which 10%, 50% and 90% of the droplet volume lie, respectively. A smaller Span value indicates a narrower droplet-size distribution [51].

#### 2.5.3. Microscopy

Emulsion microstructure was observed in bright-field mode using a conventional inverted optical microscope (CKX41, Olympus, Tokyo, Japan) at 20× magnification [48]. Samples were diluted 6-fold with deionized water to separate overlapping droplets and thereby better resolve the droplet-size distribution and the extent of aggregation.

#### 2.5.4. Rheological Measurements

Rheological properties were evaluated using a rotational rheometer (ARES-G2, TA Instruments, New Castle, DE, USA) with a parallel-plate geometry (40 mm diameter, 500 μm gap) [50]. Apparent viscosity was measured at shear rates from 0.1 to 100 s^−1^. Dynamic oscillatory frequency sweeps were conducted from 0.1 to 100 rad/s at 1% strain (within the LVR) at 25 °C [52].

#### 2.5.5. The Creaming Index

The grayscale values of the images were obtained using ImageJ (version 1.54g). The creaming index (CI%) was calculated as:

(3) CI% = (Hs/Ht) × 100

where Hs is the height of the serum layer and Ht is the total height of the emulsion; both heights were measured from the appearance images using ImageJ.

### 2.6. Storage Stability

Fresh emulsions were transferred to sealed glass vials (10 mL) and stored at 4 °C for 14 days. Visual appearance was recorded by digital photography at 1, 7, and 14 days [43]. At 1 and 7 days of storage, the droplet size and ζ-potential of the stored emulsions were re-determined following Section 2.6. The volume-weighted mean diameter (D_4,3_) was used to monitor droplet-size changes during storage, because D_4,3_ is more sensitive than D_3,2_ to the enlargement of droplets caused by coalescence.

### 2.7. In Vitro Gastrointestinal Digestion (INFOGEST 2.0)

In vitro digestion followed the standardized static INFOGEST 2.0 protocol [52]. The emulsion (oil phase 70%) was diluted to 10% oil (total volume 10 mL) and mixed with 10 mL simulated gastric fluid (SGF) containing pepsin (2000 U/mL final). The pH was adjusted to 2.0 ± 0.05 and the mixture was incubated at 37 degrees C with shaking at 200 rpm for 1 h in an incubator shaker (ZWY-110X50, Shanghai Zhicheng, Shanghai, China).

For the intestinal phase, the gastric digesta (20 mL) was mixed with 20 mL simulated intestinal fluid (SIF) containing pancreatin (100 U/mL), pancreatic lipase (2000 U/mL), and bile salts (5 mg/mL). The pH was maintained at 7.0 ± 0.05 by automatic titration with 0.5 mol/L NaOH using a pH-stat titrator (Metrohm 907 Titrando, Herisau, Switzerland) for 120 min at 37 degrees C [53]. The volume of NaOH consumed was recorded every 20 min, and the FFA release (%) was calculated. A blank digestion with deionized water replacing the emulsion was run in parallel.

### 2.8. Microscopic Observation After Digestion

The microstructure of the emulsions at the different digestion stages (initial, post-gastric after 1 h, and post-intestinal after 2 h) was observed using the same microscope (CKX41, Olympus, Tokyo, Japan) and the same 6-fold dilution at 20× magnification [54].

### 2.9. Statistical Analysis

One-way analysis of variance (ANOVA) was performed separately for each measured response (chemical composition, contact angle, WHC, OHC, EAI, D3,2, and ζ-potential), with milling time or SM-BIDF concentration as the single fixed factor; Duncan’s multiple range test was used for post hoc pairwise comparisons because it is well suited to comparing several treatment means of similar sample size. A *p*-value < 0.05 was considered significant [44].

## 3. Results

### 3.1. Structural and Physicochemical Properties of Ball-Milled BIDF

The chemical composition and dietary fiber content are shown in Table 2. N-BIDF contained 77.80% IDF (SDF not detected). With increasing milling time, IDF decreased from 77.80% to 71.48%, while SDF increased to 3.01% at 12 h, indicating partial IDF-to-SDF conversion through mechanical scission [31]. Moisture increased from 5.59% to a peak of 7.79% at 9 h, reflecting enhanced hydration of the roughened, high-surface-area particles, followed by a slight decline at 12 h [39]. Protein content remained 14.45–15.21% across treatments without clear trends.

SEM images showed N-BIDF as long fibrous bundles (several hundred μm) with smooth surfaces (Figure 1a). At 3 h, surfaces roughened and edges fragmented. At 6 h, short rods (10–30 μm) mixed with granular fragments predominated. By 9 h, sub-10 μm granular and short-rod particles dominated with a narrow size distribution. At 12 h, ultra-fine particles agglomerated into loose clusters (20–50 μm).

XRD patterns of all BIDF samples showed characteristic diffraction peaks at approximately 2θ = 15.5, 16.5, 22.5, and 34.5 degrees, corresponding to the (1–10), (110), (200), and (004) crystallographic planes of cellulose I (Figure 1b). The crystallinity index (CI) showed a trend of increasing initially and then stabilizing.

FTIR spectra confirmed that ball milling did not alter the fundamental chemical structure of BIDF (Figure 1c). All samples displayed the characteristic lignocellulosic absorption bands: a broad band at approximately 3400 cm^−1^ assigned to O–H stretching vibrations of cellulose, hemicellulose, and adsorbed water; bands at approximately 2920 and 2850 cm^−1^ corresponding to C–H stretching; a band at approximately 1620 cm^−1^ attributed to absorbed water; a band at approximately 1510 cm^−1^ arising from aromatic C=C stretching of lignin; and a band at approximately 1050 cm^−1^ corresponding to the C–O–C skeletal vibration of cellulose [55,56]. Peak positions remained unchanged across all milling durations, consistent with previous reports that ball milling and other mechanical size-reduction treatments disrupt the fiber matrix without generating new functional groups [57]. A progressive increase in the relative intensity of the ~1050 cm^−1^ band was observed with longer milling time, indicating increased surface exposure of cellulose following mechanical disintegration [39].

Ball milling progressively reduced BIDF from long fiber bundles to sub-10 μm granular particles with a narrow size distribution at 9 h. At 12 h, ultra-fine particles agglomerated into loose clusters. The cellulose I crystalline structure and chemical integrity were preserved throughout milling. A partial IDF-to-SDF conversion was observed, with SDF reaching 3.01% at 12 h. These results indicate that 9 h of ball milling achieves optimal particle size reduction.

### 3.2. Wettability and Hydration Properties

The three-phase contact angle of BIDF increased progressively with ball-milling time (Figure 2a). N-BIDF exhibited a theta of approximately 98°, indicating a slightly hydrophobic surface. As milling progressed, theta increased to approximately 100° at 3 h and further to approximately 103° at 6 h, before plateauing at approximately 105° at 9 and 12 h. This plateau suggests that the surface hydrophobicity reached saturation after 9 h of milling, and extended milling provided no additional improvement in wettability.

In contrast, both WHC and OHC decreased continuously with increasing milling time (Figure 2b). N-BIDF displayed the highest WHC (6.84 g/g) and OHC (2.36 g/g), attributable to its intact porous fibrous network that physically entraps water and oil molecules within capillary spaces and interfibrillar voids. After 12 h of milling, WHC dropped to 4.93 g/g and OHC to 1.42 g/g, representing reductions of approximately 28% and 40%, respectively. This decline reflects the progressive destruction of the fiber network architecture: as long fibers are fragmented into fine particles, the physical spaces responsible for liquid retention are substantially diminished.

Based on the wettability and hydration results, the contact angle reached a plateau at approximately 105° at 9 h, approaching a surface wettability suitable for adsorption at the oil–water interface, while approximately 65–70% of the native WHC/OHC was retained. Longer milling (12 h) caused further loss of hydration capacity without additional improvement in wettability. Therefore, BIDF milled for 9 h (SM-BIDF) was selected for subsequent emulsion preparation, representing the optimal balance between enhanced interfacial properties and preserved bulk functionality.

### 3.3. Emulsion Formation: BIDF-Only vs. SPI-BIDF Composite

BIDF alone produced no stable emulsions at any tested oil volume fraction (0.1–0.9) or milling time (Figure 3a). Within 1 h, all BIDF-only samples exhibited nearly complete phase separation.

When SM-BIDF was combined with SPI (2% *w*/*v*), stable emulsions formed across all tested oil fractions (0.1–0.9). The optimal visual stability after 24 h was observed at φ = 0.7, where phase separation was minimal across the full SM-BIDF concentration range (Figure 3b). To substantiate these visual observations, the creaming index (CI%) and image grayscale of each emulsion were quantified. Within each oil fraction, the CI% was averaged over the six SM-BIDF concentration groups (0, 0.05, 0.1, 0.2, 0.3, and 0.4%), and the grayscale was compared across the same six groups. The CI% reached a minimum at φ = 0.7 among all oil fractions tested, confirming that this formulation exhibited the least phase separation and thus the greatest stability. Grayscale analysis of the appearance images provided a complementary colorimetric assessment; grayscale values were similar across most oil fractions, except for a higher value at φ = 0.9, which reflects the denser, more opaque and concentrated emulsion at this very high oil fraction rather than superior stability. Because the grayscale metric was less sensitive to the degree of phase separation than the CI%, the CI% was adopted as the primary quantitative index of stability. These quantitative results corroborate that φ = 0.7 is the optimal oil phase fraction, and all subsequent experiments were therefore performed at φ = 0.7.

### 3.4. Emulsifying Activity, Droplet Size, and ζ-Potential

EAI, ζ-potential, D_3,2_ and Span index exhibited a non-monotonic dependence on SM-BIDF concentration (Figure 4a–c). All emulsions exhibited an absolute ζ-potential above approximately 35 mV; from an electrostatic point of view, therefore, all samples possessed a stable interface, since food systems with |ζ|-potential higher than 30 mV are generally considered electrostatically stable [58]. Within this overall stability, the pure SPI emulsion (0% BIDF) showed the lowest EAI, the largest droplets (D_3,2_ = 18.4), and the broadest droplet-size distribution. As the SM-BIDF concentration increased from 0 to 0.1%, EAI reached a maximum of 2.42 m^2^/g; D_3,2_ and the Span index decreased to minima of 8.68 μm and 1.93, respectively; and |ζ|-potential peaked at 53.23 mV. Above 0.1%, EAI declined, D_3,2_ and the Span index increased again, and |ζ|-potential decreased toward 35–40 mV, indicating that excess fiber particles compromised the interfacial integrity and reduced the electrostatic stabilization, although the interface remained electrostatically stable. Finally, it should be noted that, for this system, the droplet size measured by laser diffraction is affected by the fiber particles, which cannot be fully distinguished from the oil droplets; the reported values should therefore be regarded as apparent.

### 3.5. Microstructure, Rheological Behavior, and Storage Stability

The trends in droplet size and extent of aggregation observed by microscopy were consistent with those in the ζ-potential data: the concentration at which the smallest and most uniform droplets were observed (0.1% SM-BIDF) was also that at which the absolute ζ-potential was highest (Figure 5a). The pure SPI emulsion showed a broad droplet-size distribution with visible coalescence. At 0.05% SM-BIDF, droplets were visibly smaller and more uniform. At 0.1% SM-BIDF, the field was densely packed with fine, homogeneous droplets and negligible coalescence. At 0.2% and above, localized droplet aggregation and large coalesced domains became increasingly prevalent.

All emulsion samples exhibited shear-thinning behavior, with apparent viscosity decreasing with increasing shear rate from 0.1 to 100 s^−1^ (Figure 5b). The 0.1% SM-BIDF emulsion showed the highest apparent viscosity at all shear rates. At higher fiber loadings (0.2–0.4%), viscosity values fell between the 0.1% and SPI-only values.

All emulsions exhibited G′ > G″ over the tested frequency range, with only a weak frequency dependence, indicating that the systems behaved as weak, structured materials (weak gels). This is consistent with the general rheological behavior of food emulsion gels based on plant proteins, polysaccharides and fibers, in which the network is held together by physical (non-covalent) interactions rather than chemical cross-links [40,59]. The emulsion containing 0.1% SM-BIDF showed the highest G′ magnitude, reflecting a denser continuous-phase network, whereas the slopes of the G′ curves were similar among the samples, indicating that the fiber concentration modulated the magnitude rather than the nature of the network.

The pure SPI emulsion phase-separated within 1 day at 4 °C. By day 7, a pronounced aqueous layer had formed at the bottom, and by day 14, an oil layer was visible at the top alongside the serum phase (Figure 6). The 0.05% SM-BIDF emulsion formed a self-supporting gel within 1 day, though a thin serum layer appeared by day 14. The 0.1% SM-BIDF emulsion remained fully homogeneous throughout the entire 14-day test (no creaming, no serum, no oiling-off). At 0.2–0.4% SM-BIDF, emulsions remained stable for approximately 7 days but developed a serum layer between days 7 and 14.

To corroborate the visual observations with quantitative physical measurements, the droplet size (D_4,3_) and ζ-potential of the stored emulsions were monitored at 1 and 7 days (Figure 6). The 0.1% SM-BIDF emulsion showed the smallest change in both D_4,3_ and |ζ|-potential over storage indicating negligible droplet coalescence and sustained electrostatic stabilization. In contrast, the SPI-only emulsion exhibited a marked increase in D_4,3_ and a decline in |ζ|-potential within days, reflecting progressive coalescence, while the 0.2–0.4% formulations showed intermediate changes, consistent with their partial phase separation. These quantitative trends corroborate the visual observations and confirm that the 0.1% SM-BIDF emulsion conferred the greatest storage stability.

### 3.6. In Vitro Gastrointestinal Digestion

FFA release curves for all emulsions showed a biphasic pattern: a rapid phase during the first 40 min, followed by a slower, sustained release (Figure 7a). The SPI-only emulsion showed the fastest and most extensive lipolysis, whereas the addition of SM-BIDF reduced both the rate and extent of FFA release at all concentrations tested. The strongest inhibition was observed at 0.1% SM-BIDF, which exhibited the slowest FFA release rate and the lowest final release at 120 min (24.76%). At 0.05%, the final release was higher than that at 0.1%, and at 0.2–0.4%, the inhibitory effect progressively diminished, indicating a loss of the digestion barrier at supra-optimal fiber concentrations.

After gastric digestion, the SPI-only emulsion showed extensive droplet fragmentation (Figure 7b). The 0.1% SM-BIDF emulsion largely retained droplet integrity, with only minor edge roughening. After intestinal digestion, SPI-only droplets were nearly completely digested, leaving only residual particles. The 0.1% SM-BIDF emulsion still contained recognizable droplets of reduced size. At 0.2–0.4%, post-intestinal images showed a mixture of partially digested and intact droplets.

## 4. Discussion

### 4.1. Ball Milling as a Structure-Engineering Tool for IDF

The morphological evolution from fiber bundles to sub-10 μm particles (Figure 1a) aligns with reports on ball-milled IDF from wheat bran [39], pomelo peel [48], and bamboo culm sheath [25]. The CI showed a two-phase trend: a slight increase at 3 h, followed by stabilization with prolonged milling time (Figure 1b). This is characteristic of the mechanical action of ball milling on semicrystalline cellulose, where mechanical energy preferentially disrupts the more loosely packed amorphous regions before affecting the crystalline domains [60]. Mechanical size reduction is known to shift part of the IDF toward the soluble fraction. Ball milling converted part of the IDF into SDF in rice bran [61] and produced a redistribution from the insoluble to the soluble fiber fraction in orange peel and wine grape pomace [57]. In the present study the SDF content increased from not detectable in N-BIDF to 3.01% after 12 h of milling. This is consistent in direction with the above reports, although the absolute increase was smaller than in matrices with a higher initial hemicellulose content; this is attributable to the relatively low hemicellulose fraction of bamboo culm tip IDF. FTIR (Figure 1c) confirms purely physical modification—no new peaks or shifts—with the intensified ~1050 cm^−1^ band supporting increased cellulose surface exposure [38]. The theta shift from ~98 to ~105 degrees (Figure 2a) places SM-BIDF near the optimal range for adsorption at the O/W interface [62], attributed to preferential hemicellulose removal and exposure of less-hydroxylated cellulose crystallite faces [63]. The direction of the WHC/OHC (Figure 2b) change depends on the intensity of the mechanical treatment. Mild ball milling can increase WHC and OHC, as reported for Polygonatum IDF (WHC 5.12 g/g, OHC 2.83 g/g) [34] and for sea buckthorn IDF, in which WHC, OHC and swelling capacity increased by 16.13%, 14.29% and 15.38% after ball milling combined with cellulase treatment [64]. When the treatment is more intense, the opposite behavior is observed: super-fine grinding significantly decreased WHC, oil-binding and swelling capacities in wine grape pomace DF [65], and excessive grinding of asparagus pomace likewise reduced WHC, oil-binding and swelling capacities [66]. Our results follow the second pattern. Mechanistically, the prolonged milling reduced the fiber to sub-10 μm particles and partially converted IDF into SDF, which progressively destroyed the porous fibrous network and the inter-particle void volume that physically entrap water and oil; the loss of this physical entrapment capacity outweighs the increase in specific surface area [35].

### 4.2. SPI-BIDF Synergy and Concentration-Dependent Stabilization

Native and ball-milled BIDF alone could not stabilize emulsions (Figure 3a), consistent with reports that IDF particles, despite moderate hydrophobicity, lack the rapid adsorption kinetics for coherent interfacial films [67]. Combining SM-BIDF with SPI produces a synergistic effect: SPI provides rapid adsorption, while SM-BIDF reinforces the interfacial film and, at sufficient concentrations, the continuous-phase fiber–protein network [40,41]. Here, the network refers to a continuous-phase network formed by the non-adsorbed fiber particles (which have a broad size distribution extending into the tens of microns) together with the associated SPI, rather than to a layer of particles surrounding individual droplets. Similar synergy has been reported for CNF-SPI [43], bacterial cellulose–SPI [44], and okara IDF-SPI [45] systems. The optimal φ = 0.7 (Figure 3b) balances droplet packing with network formation; at lower fractions, the continuous phase is too dilute to form an effective network, while at higher fractions, close packing promotes coalescence [43]. The non-monotonic concentration dependence of all emulsion properties (Figure 4) is characteristic of particle-stabilized systems when loading exceeds the interfacial saturation threshold [12]. At 0–0.1% SM-BIDF, co-adsorption reinforces the interfacial film, increasing surface coverage, mechanical strength, and net negative charge density (Figure 4c), which collectively reduce droplet size and enhance coalescence resistance [50]. At 0.1%, interfacial coverage approaches saturation, and the fiber forms a percolating continuous-phase fiber–protein network (Figure 5b,c)—evidenced by near-frequency-independent G′—that physically entraps oil droplets [52]. Above 0.1%, bridging flocculation and depletion attraction [68] disrupt interfacial uniformity and degrade network integrity. The fact that 0.05% SM-BIDF already conferred inversion stability, while 0.1% was required for long-term storage stability, suggests that the network density must reach a critical level before long-term stability is achieved.

At the molecular level, the SPI–BIDF synergy is underpinned by several non-covalent interactions between the protein and the fiber surface. The abundant hydroxyl (–OH) groups of cellulose and hemicellulose, together with the polar side chains and backbone amide groups of SPI, form an extensive hydrogen-bonding network at the interface. The aromatic rings and methoxyl (–OCH_3_) moieties of lignin associate with the nonpolar amino acid residues of SPI (e.g., phenylalanine, tyrosine, and leucine) through hydrophobic interactions, whereas the partially ionized carboxyl (–COOH) groups of fiber-associated uronic acids interact electrostatically with the charged residues of the protein [69]. Collectively, these interactions anchor SPI onto the fiber and densify the composite interfacial film [70]. This interpretation is consistent with the stabilization reported for bacterial cellulose–SPI [44], cellulose nanocrystal–SPI [67], and okara IDF–SPI Maillard conjugates [45] systems.

### 4.3. Implications for Lipid Digestion Control

The observation that all SM-BIDF-containing emulsions released less FFA than the SPI-only control (Figure 7a) confirms that the composite interface provides a consistent barrier against lipolysis. The strongest inhibition at 0.1% SM-BIDF (24.76% FFA at 120 min) is consistent with two concurrent mechanisms: (i) the dense SPI-BIDF interfacial film sterically hinders lipase adsorption and bile salt displacement, and (ii) the continuous-phase fiber–protein network restricts enzyme access to confined oil droplets [54,71]. This dual-barrier effect is consistent with previous reports on fiber-network-stabilized emulsion, where interfacial coverage and network formation were identified as key modulators of lipolysis [72]. Both barriers are optimal at the same concentration because interfacial saturation and percolating network formation converge near 0.1%. Above 0.1%, partial loss of interfacial integrity, as evidenced by increased droplet size and reduced |ζ|-potential, permits more efficient lipolysis by creating regions of thin or partially uncovered interface [71]. Post-digestion microscopy (Figure 7b) supports this interpretation: 0.1% SM-BIDF droplets remained largely intact after gastric digestion and showed only partial intestinal degradation, whereas SPI-only droplets are rapidly digested.

At the molecular level, the barrier function described above has a clear chemical basis. FTIR confirmed that the milled fiber retains abundant hydroxyl groups (O–H band at approximately 3400 cm^−1^) and lignin aromatic moieties (C=C at approximately 1510 cm^−1^) (Figure 1c). These hydroxyl groups form hydrogen bonds with the amide and carbonyl groups of SPI, whereas the lignin aromatic rings engage in hydrophobic contacts with the nonpolar side chains of the protein, as discussed in Section 4.2. Acting together, these interactions create a densely cross-linked interfacial film that bile salts must first displace and lipase must penetrate before lipolysis can proceed [73]. Because bile salts displace interfacial material mainly through hydrophobic insertion and lipase adsorption requires accessible interfacial hydrophobic patches, the tightly associated SPI–BIDF film reduces both the ease of displacement and the number of available adsorption sites [12]. This chemical interpretation is supported by reports that cellulose–SPI interfaces, which rely on the same hydrogen-bonding and hydrophobic interactions, likewise retard lipolysis [74]. Thus, the convergence of interfacial saturation and percolating network formation at 0.1% SM-BIDF simultaneously maximizes the physical and chemical barrier, accounting for the lowest FFA release observed at this concentration.

### 4.4. Practical Implications and Future Perspectives

The intended application of the milled bamboo culm tip IDF is as a low-cost, clean-label structuring ingredient for oil-in-water food emulsions [75], and the resulting fiber-network-stabilized (emulgel-type) emulsions are intended as delivery systems for lipophilic nutrients with a controllable rate of lipid digestion. Valorizing bamboo culm tips as bio-based structuring agents aligns with circular bioeconomy principles. Bamboo is a fast-growing renewable resource with large processing volumes in Asia. However, planetary ball milling is energy-intensive, and industrial scale-up requires techno-economic evaluation. The narrow concentration window (~0.1%) of the system demands precise formulation control during manufacturing. Future work should validate the in vivo relevance of the lipid digestion modulation observed here, assess the emulsion as a delivery vehicle for lipophilic bioactives, and quantify the effects of ball milling on specific chemical components (e.g., hemicellulose and lignin content) to better understand the structural basis of the observed functional changes [71].

### 4.5. Limitations

Several limitations of this study should be noted. The effects of ball milling on the quantitative composition of hemicellulose and lignin fractions were not determined. The in vitro digestion employed a static protocol (INFOGEST 2.0); dynamic digestion models or in vivo studies are needed for physiologically relevant assessment. The application of the SPI–BIDF emulsion as a delivery vehicle for bioactive compounds remains unexplored. Moreover, it should be emphasized that the present study is a laboratory-scale proof-of-concept and has not yet been scaled to the industrial level. The formulation window is relatively narrow and requires precise control of fiber loading, while the current planetary ball-milling step is batch-wise and energy-intensive; these aspects, together with milling-media wear and potential contamination, need to be addressed through pilot-scale formulation and process optimization before industrial application can be considered.

## 5. Conclusions

This study demonstrated that ball milling is an effective structure-engineering strategy for converting bamboo culm tip insoluble dietary fiber into fine particles suitable as a structuring agent for oil-in-water emulsions, while preserving the cellulose I crystal form and the main chemical structure. Combined with soy protein isolate, the ball-milled fiber yielded oil-in-water fiber-network-stabilized (emulgel-type) emulsions that neither component alone could stabilize. A moderate fiber loading produced a dense composite interfacial film together with a continuous-phase fiber–protein network, which jointly conferred excellent physical stability and pronounced retardation of in vitro lipid digestion; this behavior was rationalized by a concentration-dependent mechanism of interfacial reinforcement, network optimization, and excessive weakening. Overall, this work establishes a sustainable route to valorize bamboo culm processing waste into a clean-label, dual-functional co-stabilizer for emulsions with tunable lipid digestibility.

## Figures and Tables

**Figure 1:**
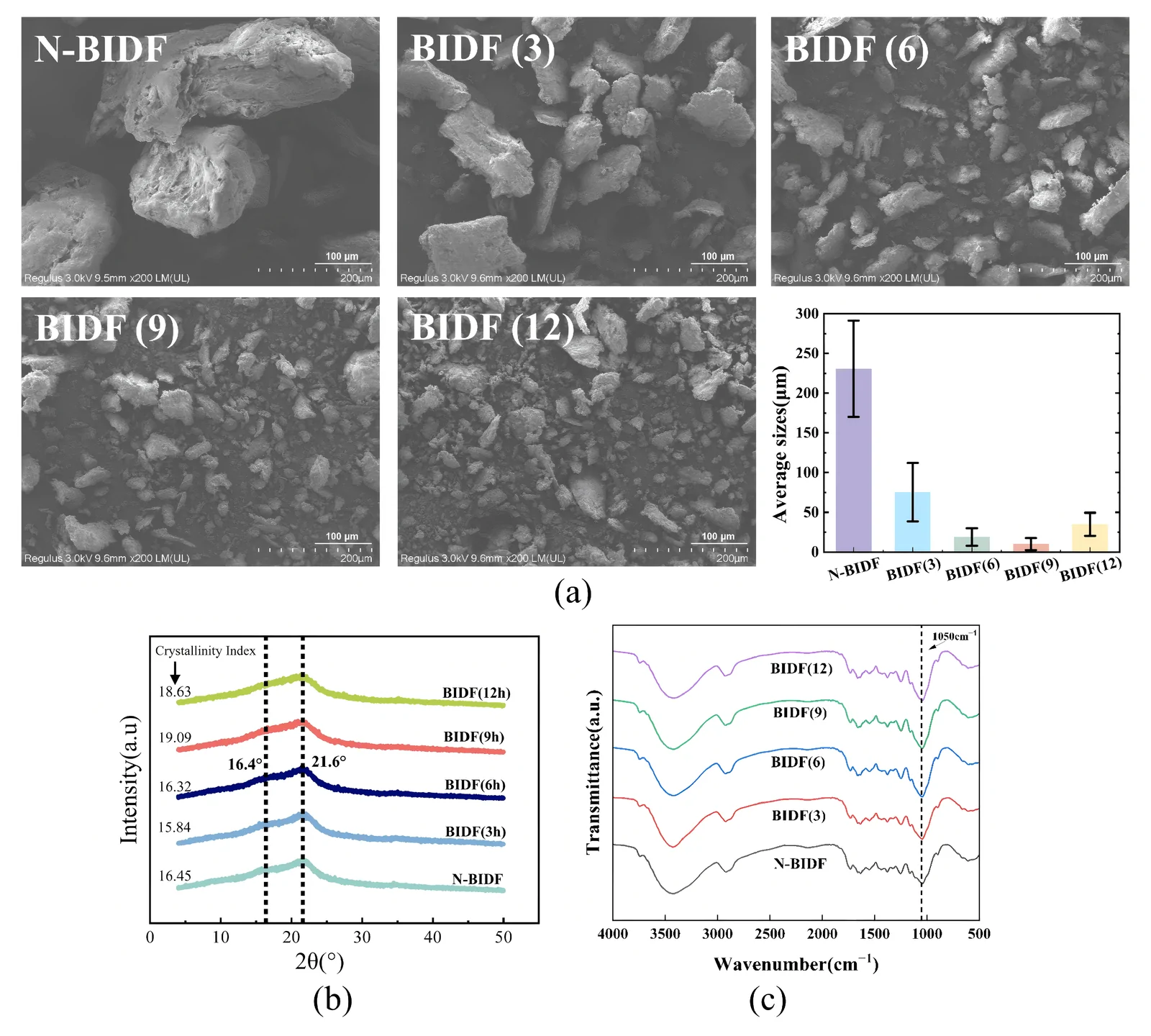
(**a**) Representative SEM micrographs of BIDF treated by ball milling for different durations (N-BIDF and BIDF (3, 6, 9, 12 h)), with scale bars indicated, together with the average particle size as a function of ball-milling time determined from the SEM micrographs using ImageJ (n = 10–50 particles per sample); (**b**) XRD patterns of BIDF treated by ball milling for different durations; (**c**) FTIR spectra of BIDF treated by ball milling for different durations.

**Figure 2:**
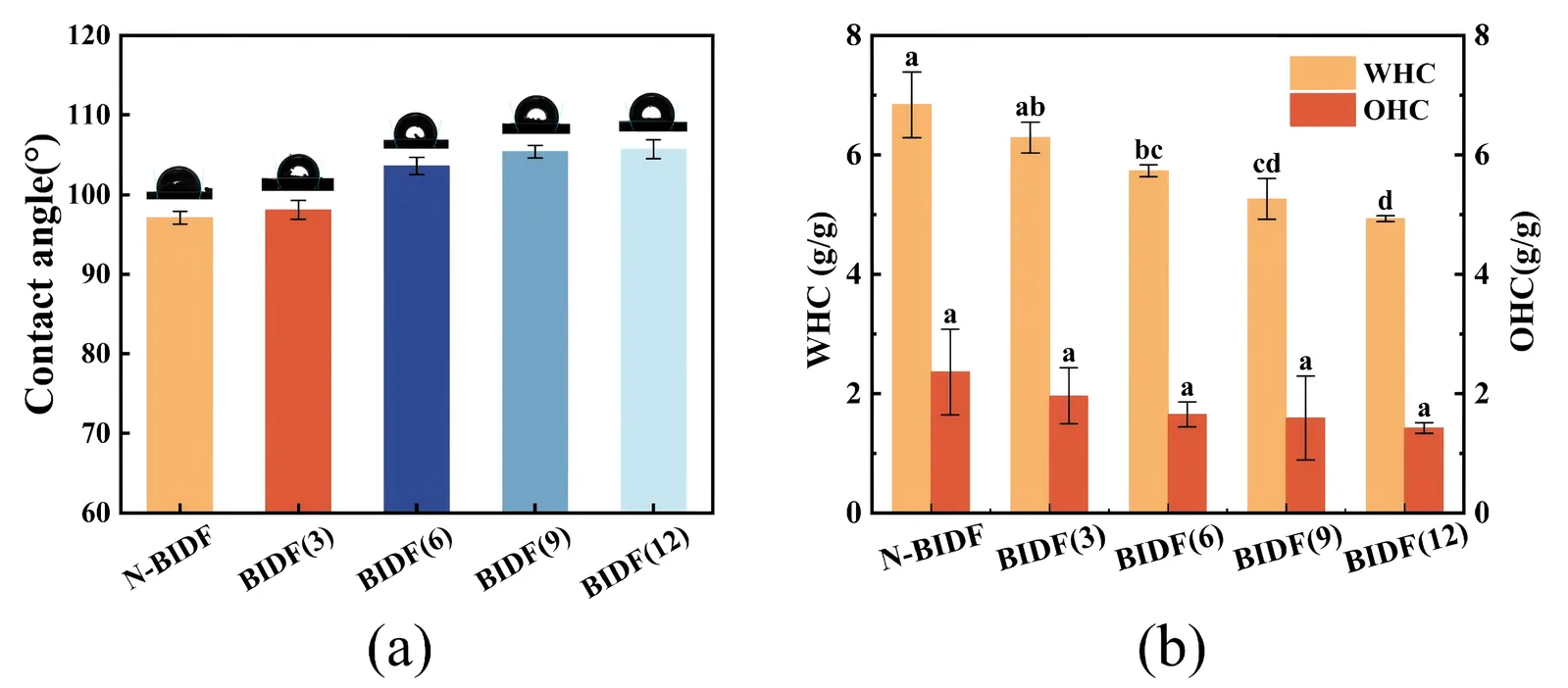
(**a**) The contact angles of BIDF treated by ball milling for different durations (N-BIDF and BIDF (3, 6, 9, 12 h)); (**b**) the water-holding capacity (WHC) and oil-holding capacity (OHC) of BIDF as a function of ball-milling time. The different letters (a–d) within same index are significantly different at *p* < 0.05. All reported values are the means of three independent replicates, with error bars representing the standard deviation (SD).

**Figure 3:**
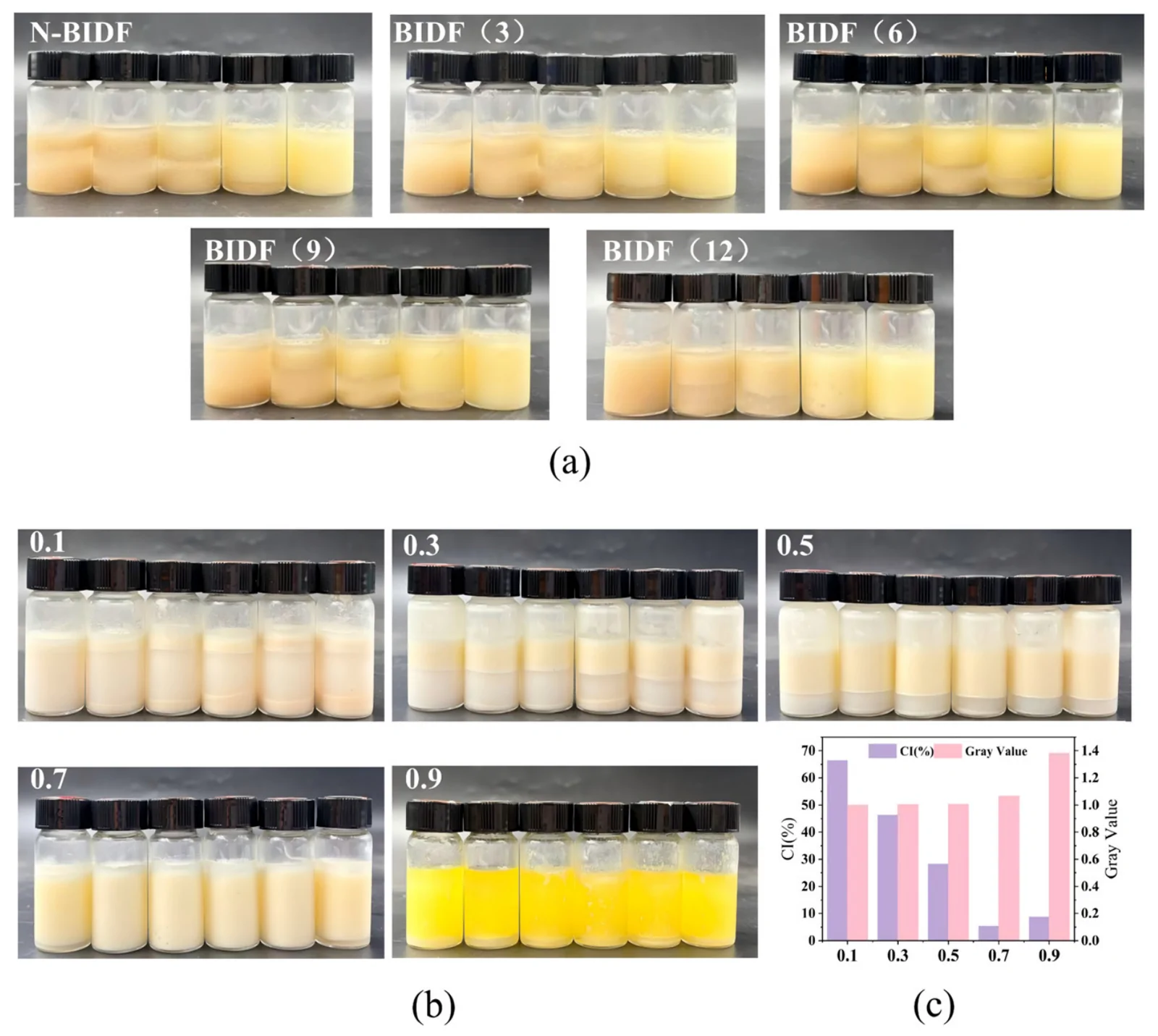
(**a**) The appearance of the emulsions prepared with BIDF alone at different oil phase volume fractions; the samples are arranged from left to right in the order φ = 0.1, 0.3, 0.5, 0.7 and 0.9; (**b**) the appearance of the SPI-BIDF composite emulsions at different SM-BIDF concentrations; the samples are arranged from left to right in the order SP-IDF0, SP-IDF0.05, SP-IDF0.1, SP-IDF0.2, SP-IDF0.3 and SP-IDF0.4; (**c**) the creaming index (CI%) and image grayscale of the SPI-BIDF emulsions as a function of oil volume fraction, where each value is the mean of the six SM-BIDF concentration groups (0, 0.05, 0.1, 0.2, 0.3, and 0.4%) within each oil fraction.

**Figure 4:**
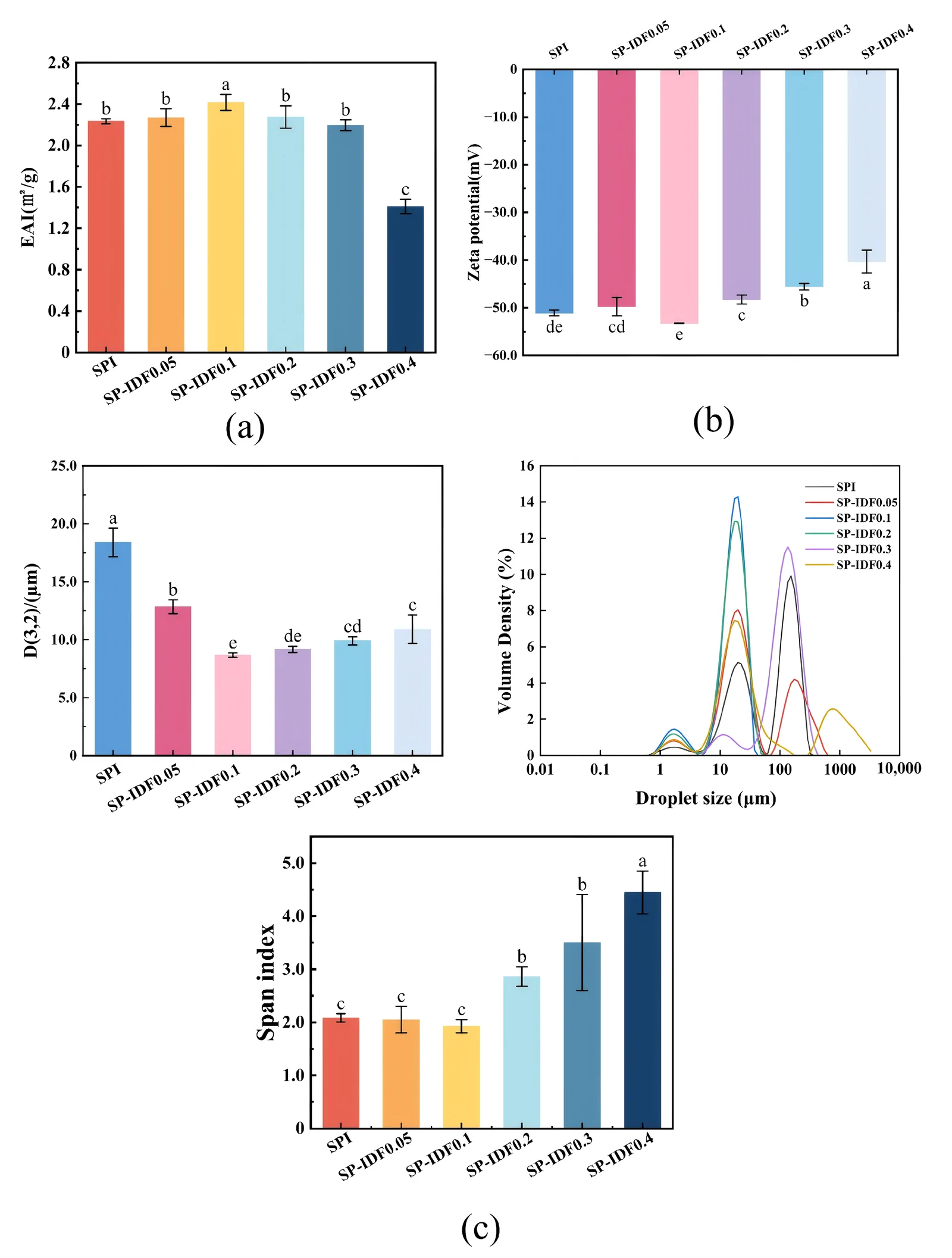
(**a**) The EAI of different SM-BIDF addition levels; (**b**) the ζ-potential of emulsions as a function of SM-BIDF concentration; (**c**) the droplet size and Span index of emulsions with different SM-BIDF concentrations. The different letters (a–e) within the same index are significantly different at *p* < 0.05. All reported values are the means of three independent replicates, with error bars representing the standard deviation (SD).

**Figure 5:**
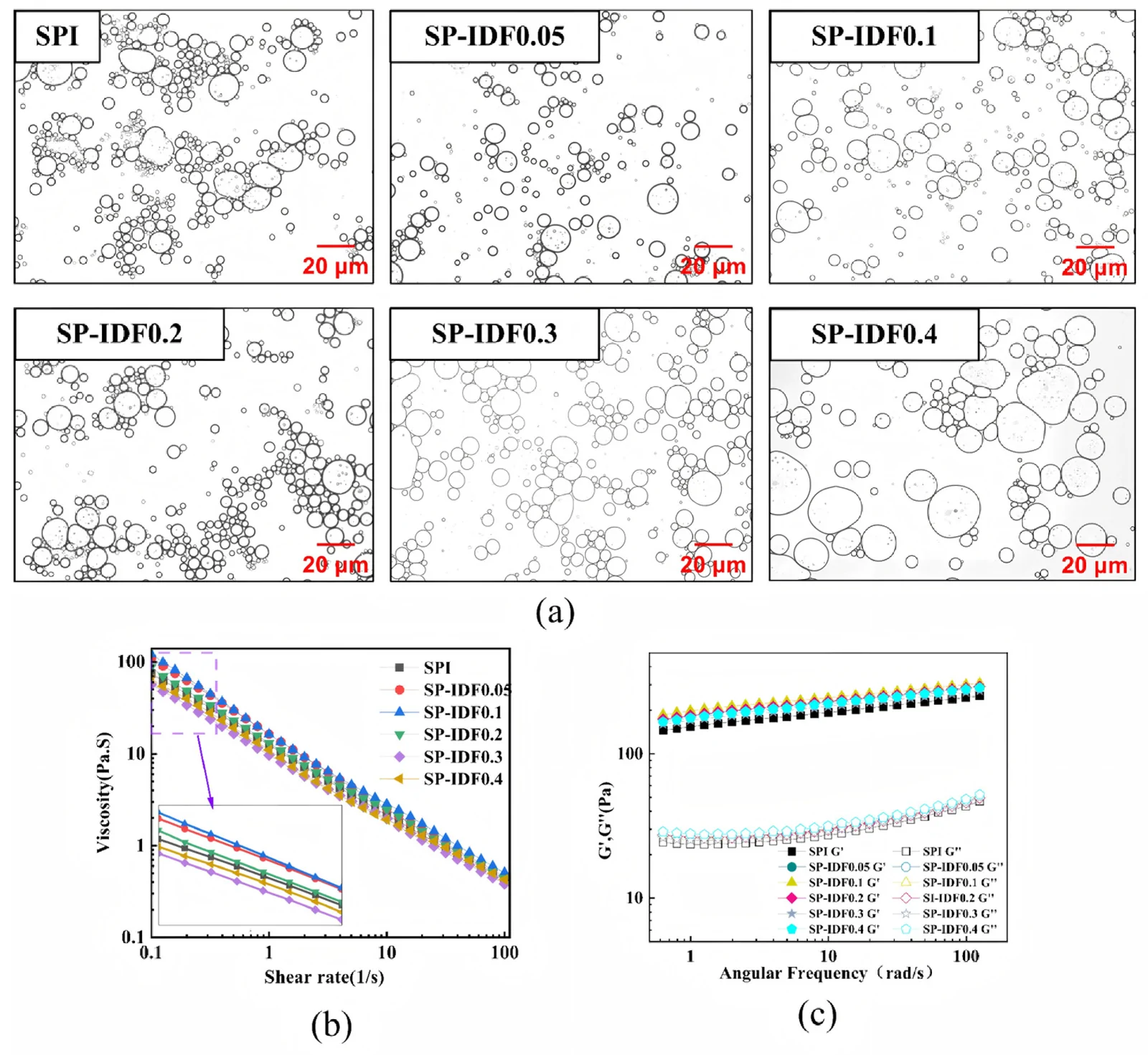
(**a**) The bright-field micrograph images as a function of different SM-BIDF concentrations; the rheological properties of emulsions as a function of SM-BIDF concentration; (**b**) the apparent viscosity vs. shear rate; and (**c**) G′ and G″ vs. angular frequency (the zoom is provided to highlight the low-shear-rate region, where differences among samples are most pronounced).

**Figure 6:**
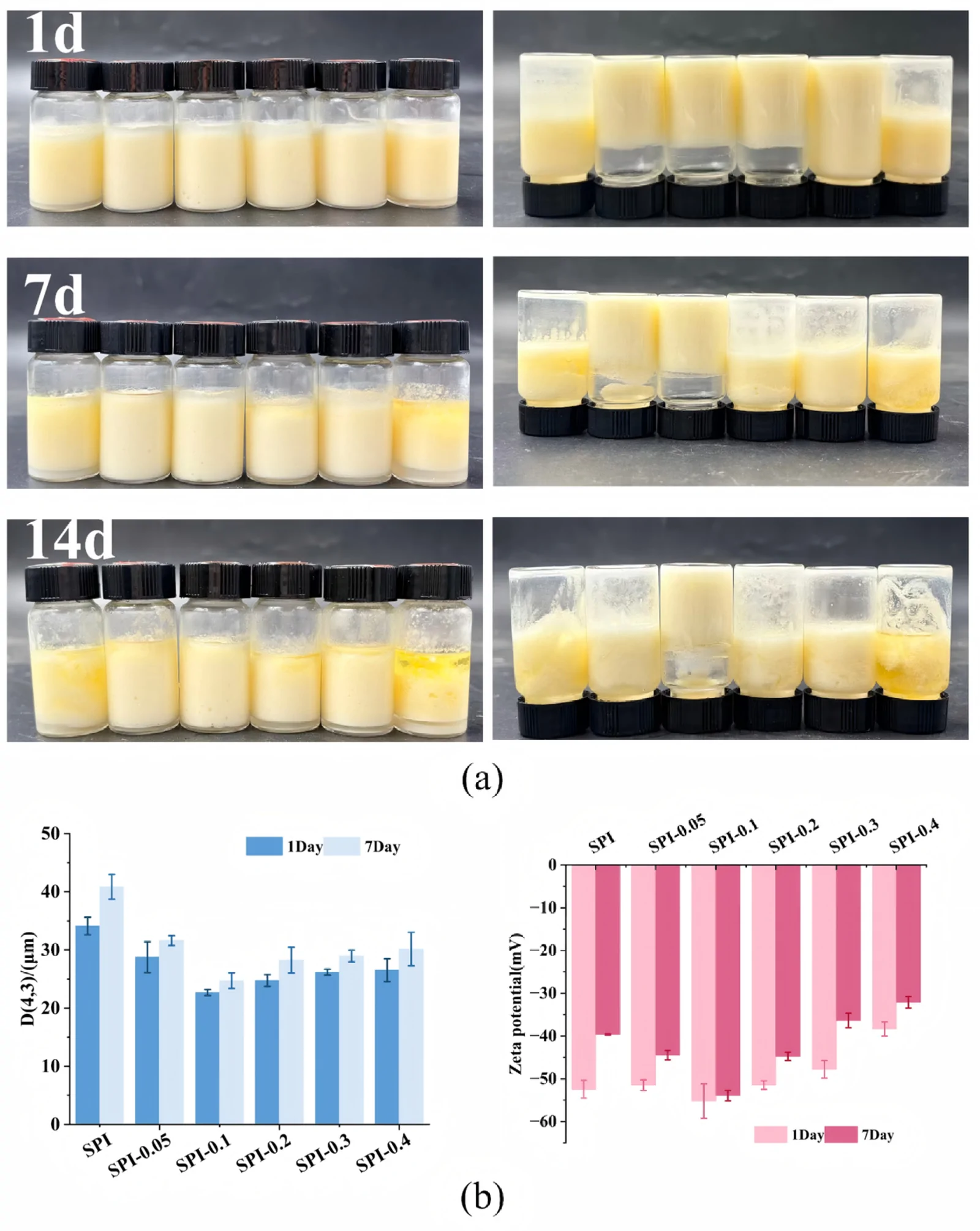
(**a**) Visual appearance at 1, 7, and 14 days; within each row (corresponding to one storage time), the samples are arranged from left to right in the order SP-IDF0, SP-IDF0.05, SP-IDF0.1, SP-IDF0.2, SP-IDF0.3 and SP-IDF0.4; (**b**) D_4,3_ and|ζ|-potential at 1 and 7 days.

**Figure 7:**
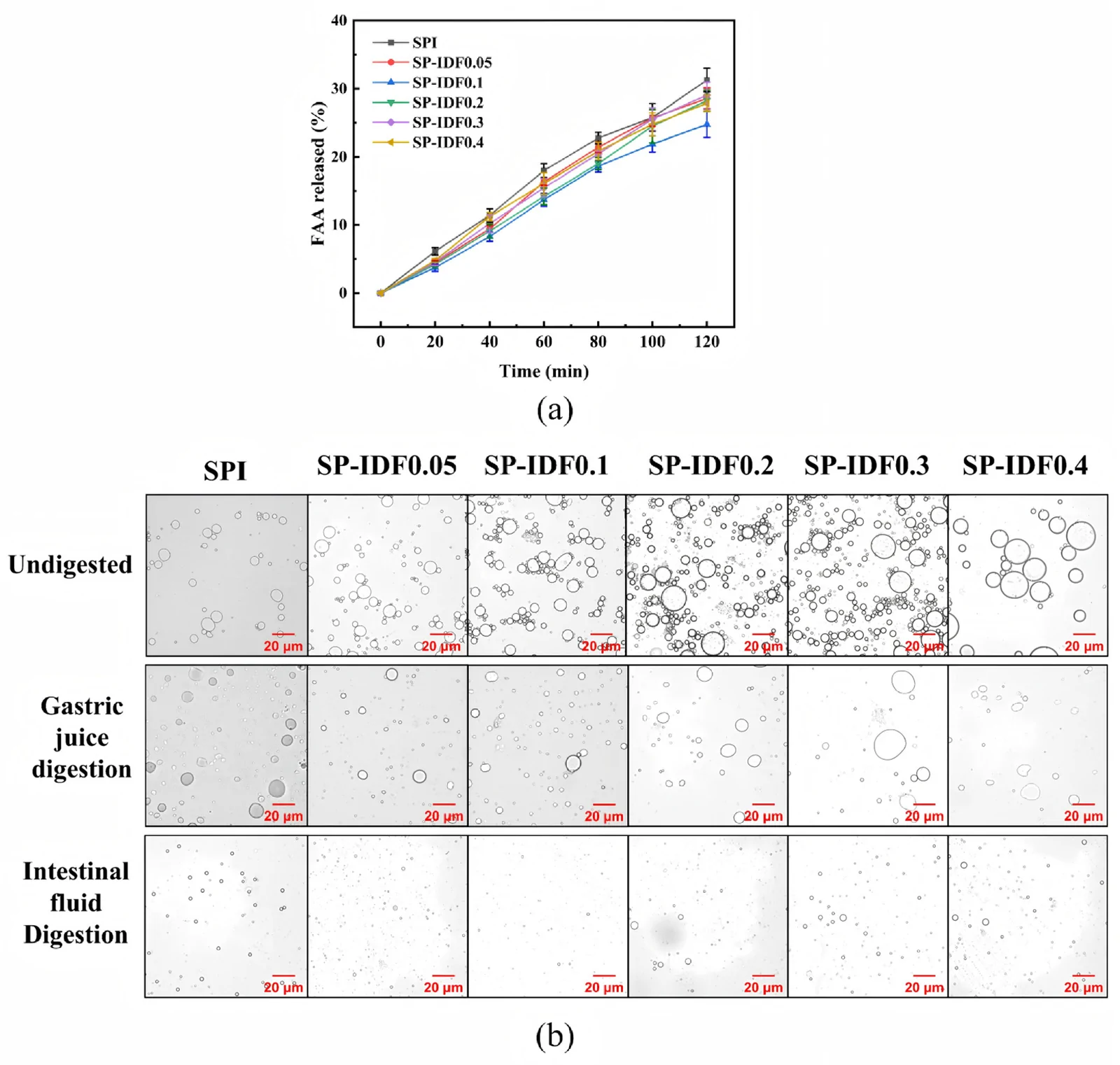
(**a**) Digestion curves of emulsions as a function of SM-BIDF concentration; (**b**) optical micrographs at the post-gastric stage (1 h) and optical micrographs at the post-intestinal stage (2 h). Scale bar = 20 μm.

**Table 1:** Sample identification and composition of SPI-BIDF emulsions.

Samples	SPI (% *w*/*v*)	SM-BIDF (% *w*/*v*)	Oil Volume Fraction (φ)
SPI	2.0	0	0.7
SP-IDF0.05	2.0	0.05	0.7
SP-IDF0.1	2.0	0.1	0.7
SP-IDF0.2	2.0	0.2	0.7
SP-IDF0.3	2.0	0.3	0.7

**Table 2:** Chemical composition and dietary fiber content of BIDF subjected to ball milling for different durations.

Sample	Moisture (%)	Lipid (%)	Protein (%)	Ash (%)	IDF (%)	SDF (%)
N-BIDF	5.59 ± 0.27 ^a^	0.08 ± 0.01 ^a^	15.21± 0.29 ^a^	1.12 ± 0.05 ^a^	77.80 ± 0.65 ^a^	ND
BIDF (3)	6.49 ± 0.09 ^b^	0.14 ± 0.19 ^a^	14.75 ± 0.37 ^ab^	1.03 ± 0.09 ^a^	75.43 ± 0.96 ^b^	1.74 ± 0.96 ^b^
BIDF (6)	7.38 ± 0.26 ^c^	0.14 ± 0.08 ^a^	14.64 ± 0.13 ^b^	1.07 ± 0.16 ^a^	74.90 ± 1.28 ^b^	2.70 ± 0.32 ^a^
BIDF (9)	7.79 ± 0.23 ^c^	0.16 ± 0.08 ^a^	14.97 ± 0.09 ^ab^	1.03 ± 0.05 ^a^	74.33 ± 0.17 ^b^	2.43 ± 0.21 ^ab^
BIDF (12)	7.41 ± 0.36 ^c^	0.17 ± 0.06 ^a^	14.45 ± 0.32 ^b^	0.97 ± 0.09 ^a^	71.48 ± 2.29 ^c^	3.01 ± 0.20 ^a^

ND: not detected. All values are expressed on a wet (as-is) basis, i.e., as g/100 g of the initial sample mass. The values are mean +/− SD (*n* = 3). Different superscript letters within a column denote significant difference (*p* < 0.05).

## Data Availability

The original contributions presented in this study are included in the article. Further inquiries can be directed to the corresponding author.
